# Addressing COVID-19 during times of competitive politics and failed institutions

**DOI:** 10.7189/jogh.11.03117

**Published:** 2021-11-13

**Authors:** Andres Barkil-Oteo

**Affiliations:** 1 Department of Psychiatry, Georgetown University School of Medicine, Washington DC, USA; 2 School of Advanced International Studies (SAIS), Johns Hopkins University, Washington DC, USA

In human history, pandemics have always had a significant impact on humans and civilizations. As shown in Thucydides' discussion on the Peloponnesian wars, a plague that spread in the region forced people to move from rural areas to the city of Athens, which led to disturbance in the balance of power and political turmoil. He even went as far as to claim that the plague changed the grand strategy of the Athenians from one of prudence to a reckless ambition that eventually triggered its decline [1]. Pandemics were also crucial (among other factors) in the Roman empire's collapse [2]. Yellow fever limited Napoleon's expansion plans in the western hemisphere out of Haiti, and Typhus devastated his Russian campaigns [1]. In another region, the native population of the Americas was wiped out not by fire alone but by the diseases brought by the Spanish explorers from Europe. In our modern history, the influenza pandemic of 1918 had devastating global effects not just because of the virulence of the disease but also because of technological and political factors, including the development of transportation, that facilitated people's movement and hence the pandemic spread. HIV/AIDS in the eighties transformed the conversation around LGBT communities and helped usher in the new security-based perspective to deal with global health and infectious diseases. In this new frame, infectious diseases are given priority due to their impact not only on health but also on security. Health issues are perceived as security threats, and nations respond to them strategically in accordance with their security agenda and not necessarily through public health focus [3].

The emergence of SARS-CoV-2 (aka COVID-19) has been one of the most critical global health events in the 21 st century. It is not just a global health issue but also a political, economic, and security problem. As of this writing, there are 233 million cases and more than 4.7 million deaths worldwide [4]. We have been through at least four pandemic waves, and the fifth one appears to be just around the corner. COVID-19 exposed the fragmentation of our globally interdependent world with its supply chain interrupted and just-in-time logistic plans collapsing. The lockdowns instituted in various countries have had severe economic effects, and the closure of borders has impacted the between-country movement of people and goods [5]. There was a sudden realization that efficient systems made countries vulnerable to sudden events like global pandemics, leading to many countries considering local production of essential goods and services.

Given the global impact of infectious diseases and the interconnected status of the world, one would imagine that the collective international response (as happened with Ebola or Polio) would be the norm, with states being interested in eradicating the virus not just within their own borders but worldwide. However, the reality has been very different, with leading nations such as the USA and China failing to provide global leadership and instead of engaging in nationalistic anti-collaborative practices. The World Health Organization (WHO), which usually provides leadership on these issues, has been bogged down with competitive politics between great powers; for example, the Trump administration withheld USA financial contributions [6], and China declined to share its data and trends on the pandemic [7]. Even integrated international political systems like the European Union (EU) witnessed the breakdown of collaboration as EU countries closed their borders to fellow citizens and refusing to offer support, which impacted EU countries [8].

**Figure:**
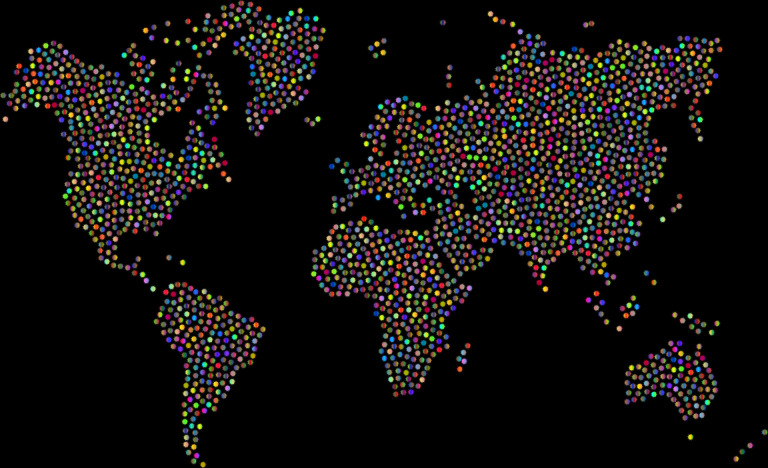
Photo: https://pixabay.com/illustrations/earth-corona-virus-globe-pandemic-5138795/.

As in many other pandemics, COVID -19 proved to be a health issue and a political problem. Political decisions have impacted country-specific responses and outcomes to the crisis irrespective of the status of their health systems. Even the most health-related technical challenges often have political ramifications; this could manifest in who is leading the response, who is consulted, and the degree of government intervention and support. For many critics, the way the WHO has managed the pandemic has ranged from being too political or not political enough to engage effectively with countries [9]. With WHO (and other global health bodies) insistence on separating the technical issues in health from the political ideologies of nations, they remain oblivious towards why countries don't collaborate even when it is in their interest to do so and what to do about it [10]. Politics cannot be set aside; even the most democratic nation can let political ideologies get in the way of effective public health response, as was demonstrated in the USA and several EU countries [11].

## LET'S GET “REAL” ON THE PANDEMIC

Since the start of the pandemic, it became apparent that nation-states were still the leading players in the international arena when it came to security and serious threats. Despite all the talk regarding the critical role of international institutions, when humanity faces existential threats (and pandemics tend to be perceived this way), people look to their national governments for security and safety. In the realist frame, cooperation between states does happen but only when it serves the nation's interest. From the moment the pandemic spread, governments engaged in competitive power politics, instituted travel bans, limits on exports, and hoarded medical supplies. Even in the EU, which is considered a model for an integrated system or society of nations, countries were quick to implement steps that violated many EU regulations, like limiting the movement of people and products across country-specific borders [5]. When Italy asked for medical supplies and resources during the peak of their epidemic, no European country was willing to donate. China took advantage of the USA's retreat from its leadership role in global health and engaged in “mask diplomacy” to strengthen its status internationally as the purveyor of public goods [12]. In addition, the breakdown and the dysfunction of world health organizations in the face of the crisis were long predicted by realist thinkers [13]. International cooperation through institutions lasts as long as it is in the interest of powerful nations. Realists are very clear that states will hesitate to collaborate due to a lack of trust in the international system, even if this was to everyone's advantage. Instead, they will focus on power politics and zero-sum games even if everyone is worse off in the long run.

Examining the COVID-19 national responses exposes the strengths and weaknesses of different types of states and regimes. Rigid dictatorships are especially vulnerable to crises like pandemics or natural disasters, primarily because of the secrecy, suppression of information, and the avoidance of upsetting the leadership with bad news. These regimes are often late to recognize the problem and to react. However, these regimes have the advantage of mobilizing resources once the leadership recognizes the issue. For example, China managed to implement massive lockdown efforts and surveillance to counter the pandemics [14]. These raise concerns of human rights abuse and fears that such practices may persist beyond the pandemic.

On the other hand, while democracies have a free flow of information and recognize their problems faster, they tend to struggle when a national response is required. The USA's difficulty in coordinating one federal response was very apparent at the beginning of the pandemic. There was a need to link different government agencies, hospital sectors, and states health departments, all with a variety of priorities, funding mechanisms, and operational concerns. The decentralized nature of the system may have an advantage for efficiencies, but it is paralyzed when responding to crises. In the end, the USA was saved by the fact that vaccines were effective, and they relied mainly on corporations (Walgreens, Walmart, etc.) for distribution and not on better coordination among government agencies (although that may have helped) [15,16].

## WILL LIBERALISM LIBERATE US FROM COVID-19?

The COVID-19 pandemic had all the elements to be an excellent liberal case for the importance of collaboration. COVID is a virus that “doesn't stop at borders”; it affects everyone and will not be eliminated until it is eradicated everywhere. In liberal theory, when states have a common interest, they should collaborate for the benefit of everyone [13]. In this scenario, states focus on the absolute gain “virus-free world” vs the relative gains “I have better control of the pandemic than you.” Given the interconnectedness of our global economy, everyone should be interested in addressing global impact issues. This cooperation could happen through international institutions, between NGOs and private entities across nations, and between individuals. More importantly, these collaborations tend to happen often between democracies [13]. Indeed, the COVID-19 pandemic has fueled large-scale collaborations among scientists across the globe. Scientific institutions shared their finding online ahead of publications, and the genome of COVID was sequenced and shared freely. Scientific competitive practices and secrecy practices were waived to collaborate on the greater good [17,18]. But when it came to political relationships, narrow national interests were put ahead of collective good, with integrated systems failing to work together. WHO, the leading international system to coordinate the global response has not fulfilled its usual role. In addition, even democracies with a good track record of collaboration resorted to competition for scarce resources; several examples include USA-Canada or inter-EU relationships.

## BILATERAL VS. GLOBAL HEALTH DIPLOMACY

As discussed above, the WHO has been receiving criticism regarding its handling of the pandemic. WHO has been accused of being too political, siding with China's efforts to hide their pandemic data, and refusing to incorporate data from Taiwan in the WHO covid pandemic response database. This is not new, as there have been past instances of countries hiding or delaying their information. Previously from China responding to SARS, Saudi Arabia responding to MERS, and more recently from Sierra Leone and Liberia responding to Ebola [19]. WHO has no power to sanction states who fail to report, and the organisation's financial situation is dependent on donations, which makes it hard to enforce any mandates. Given that WHO will always be limited by its funders and the sovereign state claims, one could argue that it cannot lead a global response in the absence of dominant power. In other words, it cannot fill the political vacuum created by the retreat of the USA. WHO is positioned as a technical body that could organize and share information and disseminate best practices and the deployment of experts, but not to coerce countries to comply with their regulations.

Although there were noticeable failures of multilateral collaboration, there has been an increase in bilateral health diplomacy. In the pandemic, China took a leading role early on with its donations of COVID tests and Personal Protective Equipment (PPE) kits; Russia later followed with distributions of their newly developed vaccine. These efforts were primarily targeted at countries where both powers have a strategic interest. In addition, there were instances of assistance in an unusual pattern, from developing to developed countries, like the Indian and Turkish donation to the USA at the height of the American Pandemic. Bilateral health diplomacy is not driven by humanitarian purposes but with an eye on improving relationships. The fragmentation in the global response and the increase in bilateral and regional collaborations (outside of international institutions) has been a hallmark of the COVID-19 response.

Is it possible that from now on, pandemics will be handled through bilateral relationships instead of global health diplomacy out of international intuitions and global participants? Given the disappointment with WHO's role, states relied on regional and bilateral health diplomacy to fill the gap. Two reasons could explain this trend: First, it is easier to coordinate health diplomacy among few countries (who may already share strategic interests or cultural factors) to contain the pandemic than relying on a global response (for example, Australia and New Zeland). And second, states may look into health diplomacy to strengthen their relationships in their foreign policy agendas [19]. The USA and China rivalry is increasing the bilateral health diplomacy trend. No one is willing to take the lead globally. For China, there is a lack of interest in their foreign policy agenda and lack of credibility internationally, and for the USA, a retreat from their global role has been due to domestic and individual leaders factors. These strategies are short-sighted as a pandemic on a global scale will not be addressed unless a worldwide response is in place. It will be the difference between *eradicating* the virus and *living with* the virus.

## COLLECTIVE ACTION PROBLEM IN GLOBAL HEALTH

Since the impact is global, states should be concerned about the absolute gain when it comes to public health. However, as in many instances where public goods are provided, collective action problems arise. It is difficult to convince countries to contribute if they will benefit for free from a global decrease in infection rate, creating the free-rider problem. This issue often leads to the under-provision of services [20]. One way to cooperate in this setting is for a hegemon or a dominant power to provide the public good for free for everyone. This act could induce other countries to do the same. In global health, this was the case with HIV/AIDS. The President's Emergency Plan for AIDS Relief (PEPFAR) contributed more than 90 billion USD to AIDS treatment and provided antiretroviral therapy for millions of patients. There is currently a call to the US President to do the same for COVID-19, focusing on providing free vaccines to the world. Under the Biden administration, there has already been a contribution of more than 4 billion USD to the COVAX platform (to fund vaccine efforts in low-income countries) [21]. COVAX has an ambitious goal to deliver more than two billion doses of vaccine to developing countries. A renewed leadership role by the USA could resume nations' trust with collaboration and restart international institutions' effectiveness [21]. The USA is signaling that it is back in the international arena and leading the global health efforts as it did before with HIV and Ebola.

## CONCLUSION

Since the end of the cold war, an increasing optimism prevailed regarding the transition of the international system away from power politics towards a new era of collaboration and interdependence. However, COVID 19 showed the limits of alternative models and the resurgence of the realist view to explain the current dynamics. In global health, experts tend to overvalue the importance of collaborations and international institutions, making them unprepared for situations when alliances break down. Because of the inherent lack of trust in the system, especially in times of crisis, states return to zero-sum relationships at the expense of collaboration. If health diplomacy is changing from global to bilateral and regional relationships, it will profoundly impact our ability to combat pandemics. While an interest-driven and security lens may favor the bilateral or regional response, a narrow global health approach will make the current and subsequent pandemics last longer and have a more devastating effect. There is no replacement for a globally coordinated effort, and the WHO is in no position to do that in the absence of a dominant world power leading the response. The USA should continue to support vaccine production and distribution, which will also encourage other countries to do so. While this will be far from a comprehensive approach like that was previously used for Ebola or Polio, it may be the most feasible way forward, considering the political realities and the competitive nature of the current international system.
